# Implementing community-engaged pharmacogenomics in Indigenous communities

**DOI:** 10.1038/s41467-024-45032-5

**Published:** 2024-01-31

**Authors:** Katrina G. Claw, Casey R. Dorr, Erica L. Woodahl

**Affiliations:** 1 Department of Biomedical Informatics, Colorado Center for Personalized Medicine, University of Colorado Anschutz Medical Campus, Aurora, CO USA; 2 Hennepin Healthcare Research Institute, Minneapolis, MN USA; 3 Nephrology Division, Department of Medicine, Hennepin Healthcare, University of Minnesota, Minneapolis, MN USA; 4 Experimental and Clinical Pharmacology Department, College of Pharmacy, University of Minnesota, Minneapolis, MN USA; 5 Clinical and Translational Sciences Institute, University of Minnesota, Minneapolis, MN USA; 6 Department of Biomedical and Pharmaceutical Sciences, University of Montana, Missoula, MT USA; 7 L.S. Skaggs Institute for Health Innovation, University of Montana, Missoula, MT USA

**Keywords:** Pharmacogenomics, Ethics, Genetic predisposition to disease

## Abstract

Innovative pharmacogenomic approaches (genetic variation related to medication response) are needed to reduce disease and disparities in Indigenous communities. We support community-based pharmacogenomics research, inclusive of Indigenous values and priorities, to improve the health and well-being of Indigenous peoples.

Innovative approaches are needed to treat chronic diseases and lessen health disparities in underserved Indigenous communities, the aboriginal inhabitants of a land. The history of colonization that dislocated Indigenous peoples, and interrupted traditional lifestyles and culture, has led to enduring health disparities influenced largely by social and economic determinants of health. Additionally, development of drug treatments has generally been optimized in populations that include few, if any, Indigenous peoples. Precision medicine in Indigenous communities may offer novel strategies to close the gap in health disparities. Pharmacogenomics—a field of research that studies how genetic variation affects a person’s response to drugs—is a cornerstone of precision medicine, promising to optimize medication efficacy. A challenge for pharmacogenomics is ensuring that the benefits are distributed equitably across all populations. International efforts are ongoing to facilitate implementation of pharmacogenomics into clinical care, including publishing guidelines with pharmacogenomic-specific recommendations (https://www.pharmgkb.org/guidelineAnnotations). Genetic variation fluctuates across regions of the world—according to the ancestral origins of modern biogeographical populations—and there continues to be a lack of diversity in precision medicine and pharmacogenomic research, which has implications for who is likely to benefit from such research^1–3^. Pharmacogenetic variation across much of the world’s population is likewise not well understood, leading to questions about whether current clinical pharmacogenomics will be useful or predictive for Indigenous populations.

Globally, there are over 476 million Indigenous peoples living in 90 countries, accounting for 6.2% of the population (www.un.org/en/observances/indigenous-day). Indigenous peoples in the United States (US), specifically American Indian and Alaska Natives (AIANs), represent 2.9% (9.7 million) of the population in the most recent US Census (www.census.gov). AIANs include 574 federally recognized sovereign tribes—and more unrecognized tribes, Native Hawaiians, and Pacific Islanders—with each having distinct cultural, social, and political structures, though many values are shared. Concerns shared among Indigenous peoples about genomics research include maintaining research oversight, upholding tribal sovereignty, biospecimen and data storage/use, and culturally appropriate research^4,5^. While Indigenous values are diverse, respecting holistic relationships in health and the environment, cultural integrity, and the inclusion of Indigenous knowledge resonate with many Indigenous communities, and reflect a commitment to the collective while maintaining a respectful relationship with the land and other beings^6–9^. Unfortunately, the experiences of exploitation and unethical research practices by researchers and institutions is shared across Indigenous communities and has generated distrust for research. Practices like “helicopter research”—where researchers take genetic data without long-term relationships and commitments to communities— are harmful practices that designate researchers as untrustworthy and decrease the willingness of Indigenous communities to participate in research^10,11^. While our commentary focuses primarily on work with AIAN communities, these perspectives may also apply to many global Indigenous populations.

We advocate for community-engaged pharmacogenomics research—inclusive of Indigenous values and priorities—to increase inclusion and equity in research, and to improve the health and well-being of AIAN peoples (Fig. 1). It is important for researchers to recognize the intersectionality between health, research, and spirituality in AIAN health^9^, and using a holistic medicine approach to pharmacogenomics and precision medicine research would be appropriate. A holistic approach acknowledges that all aspects in an individual’s worldview are interconnected (e.g., physical, mental, environmental, animate, inanimate), and that an individual’s health potential is dependent on the whole system of research, healthcare, and traditional knowledge. For example, a holistic approach to pharmacogenomic research with AIAN peoples would involve not only characterizing genetic variation but would include individual or community access to specific medications, lifestyle factors, social and environmental variables, and AIAN traditional knowledge and practices as a part of the care and healing process. Many tribal clinics already implement this approach, which has led to success in medication adherence and treatment^12^. To increase pharmacogenomic knowledge and research, we recommend that the scientific community prioritize the identification and validation of genotype-phenotype associations in AIAN communities, generate reference genomes inclusive of AIAN people, create pharmacogenetic testing panels representative of AIAN variation, and increase inclusion of AIAN peoples in basic and clinical research as participants and workforce members. To engage and develop collaborative pharmacogenomic research infrastructures with AIAN communities, we suggest establishing more community-academic partnerships where research questions are aligned with the priorities of the community, supporting AIAN capacity building through education and funding, and promoting collaborations with tribal healthcare systems to implement pharmacogenomic testing.

**Fig. 1:**
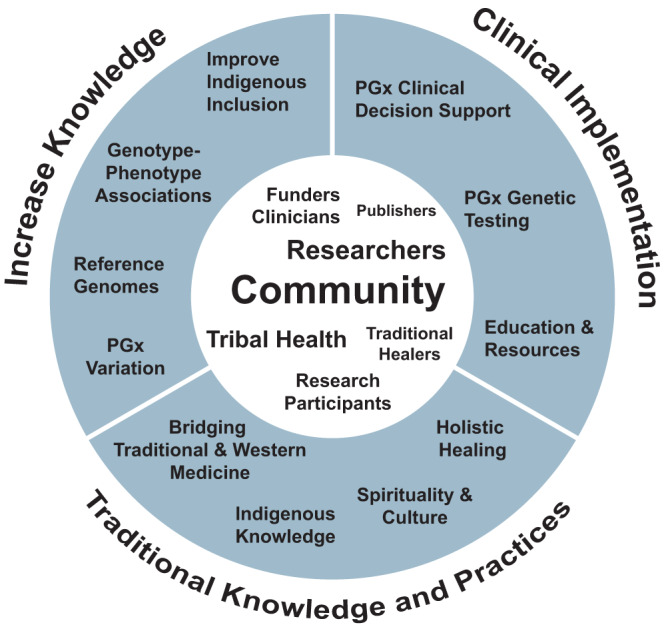
Pharmacogenomics in American Indian and Alaska Native Communities. We highlight key areas in pharmacogenomics (PGx) research and clinical implementation to bring about better health and well-being in AIAN communities: (1) Engaging the community (*center*), (2) increasing PGx knowledge of AIAN variation, (3) clinical implementation of PGx, and (4) inclusion of traditional knowledge and practices. Engaging the community is at the core, and this includes coordinating research with the tribal community, obtaining tribal approval, and encouraging tribal members to participate in data analysis, interpretation, and writing of results. Increasing knowledge related to PGx variants by including Indigenous peoples in basic and clinical research and the workforce, support for clinical implementation into healthcare, and including traditional knowledge and practices need to increase.

## Pharmacogenomic variation in Indigenous communities

Pharmacogenomic research focuses on the genetic contribution to response to medications and characterizing genetic interindividual variation in drug-metabolizing enzymes and drug transporters, which can affect drug elimination and biotransformation. Varying allele frequencies in pharmacogenes across global populations have important clinical implications, yet consistent population grouping is lacking, inadequate, or inappropriate and continues to include many racial descriptors—a topic more fully discussed in the recent National Academies report on population descriptors in genomic research^13^. The Pharmacogenomics Knowledgebase (PharmGKB) uses a biogeographic grouping system based on seven geographically defined groups, but this will have to be reassessed if data from Indigenous peoples are to be useful to distinct tribal groups, which are political, geographic, and cultural ethnic groups^14^. Increasing AIAN representation in pharmacogenomics research may lead to improved genotyping arrays that are inclusive of unique variants as well as variants that are more common in AIAN people, which may lead to improved predictions of genotype-phenotype associations. These data may lead to personalized drug therapy aimed to reduce health disparities and improve health outcomes in Indigenous people, although clearly these disparities are also impacted by health and social inequities that cannot be addressed by improved access to pharmacogenomics. Trial and error approaches traditionally used in medication management can be problematic for tribal members who may live far from healthcare facilities, and we advocate that pharmacogenomic-guided approaches be used as early as possible in prescribing practices to reduce risk of therapeutic failure. While data are sparse, there are examples where pharmacogenomics research can impact the health of AIAN peoples, including therapeutic areas of cancer^15,16^, cardiovascular disease^17^, smoking cessation^18,19^, and transplantation^20^.

Most pharmacogenetic variation remains unknown across tribes and biogeographical regions of North America. An important class of drug-metabolizing enzymes is the cytochrome P450 (CYP) gene family, which plays a pivotal role in the biotransformation and elimination of xenobiotics, including pharmaceutical compounds. We summarize the known landscape of *CYP* pharmacogenetic variability in AIAN peoples in Table 1. Notably, frequencies of *CYP* variants are highly variable and population-specific. Novel genetic variants at relatively high frequency have been identified in several *CYP* genes in AIAN populations that may result in altered enzyme activity^16–18,21^. There is a tendency to treat AIAN peoples as a homogenous group, but data from *CYP* pharmacogenes highlight the extensive heterogeneity within AIAN peoples.

**Table 1:** Cytochrome P450 (CYP) pharmacogene variation in American Indian and Alaska Native peoples

				*CYP* genes					
AIAN tribe or geographic region	*CYP2A6*	*CYP2B6*	*CYP2C9*	*CYP2C19*	*CYP2D6*	*CYP3A4*	*CYP3A5*	*CYP4F2*	*CYP4F11*
Confederated Salish and Kootenai Tribes in Montana	—	—	●^16^	—	●^16^	●^16^	●^16^	—	—
Oglala Sioux Tribe in South Dakota	—	—	—	●^42^	—	—	—	—	—
Yup’ik people in Yukon-Kuskokwim Delta, Alaska	●^43^	●^43^	● ^17^	—	—	●^21^	●^21^	●^17^	●^17^
Northern Plains Tribe in South Dakota	●^19^	—	—	—	—	—	—	—	—
Southcentral Foundation in Alaska	●^18^	●^18^	—	—	—	●^21^	●^21^	●^17^	●^17^
Southwest Tribe in Arizona	●^44^	—	—	—	—	—	—	—	—

The table was modified and updated (two additional citations^18,21^) from Henderson et al. ^45^, and includes studies that involved specific AIAN tribes and geographic regions in the United States. Studies that reported more generalized population descriptors (broad AIAN groups), non-AIAN Indigenous groups, and ancestry-informed and imputed alleles were excluded from this table. The systematic literature review inclusion criteria from Henderson et al. ^45^ was followed for the additionally included publications. ● indicates that pharmacogenomics data from those tribes/regions has been published and references are indicated in the superscript.—indicates that no data is available.

We highlight two examples where inclusion of AIAN participants in pharmacogenomics research has led to findings with important clinical implications. The first is the use of *CYP3A5* pharmacogenetic-based dosing of a primary immunosuppressant used in transplantation, tacrolimus^22,23^. Specific variants are designated by star (*) alleles, such as CYP3A5*1 or *3. *CYP3A5* variant frequencies differ by population group, with normal function CYP3A5*1 and no function CYP3A5*6 and CYP3A5*7 enriched in populations of recent African ancestry^24^, while the no function CYP3A5*3 variant is more common in most other populations. Researchers have further identified the CYP3A5*3 variant at high-frequency and the CYP3A5*6 and *7 variants at low-frequencies in three AIAN communities in Montana^16^ and Alaska^21^, similar to variant frequencies in self-identified AIAN kidney transplant recipients^20^. In transplantation, to avoid sub-therapeutic tacrolimus plasma concentrations and the potential for allograft rejection, individuals carrying a CYP3A5*1 allele require higher doses of tacrolimus compared to individuals carrying CYP3A5 *3, *6 or *7 alleles, making inclusion of AIAN in testing an important consideration. Another example of pharmacogene variation in AIAN populations is the identification of a common, novel, function-disrupting variant in *CYP2C9* called *M1L* that predicts response to the anticoagulant warfarin^17^. Further in vitro characterization found that *CYP2C9 M1L* conferred reduced catalytic activity^25^ and an in vivo pharmacokinetic study suggested that *M1L* carriers exhibited slower drug elimination^26^. For *CYP2C9* pharmacogenetic-based warfarin dosing, patients with the *M1L* variant are at risk of adverse events when given a standard dose of warfarin and may require a lower starting dose at initiation of warfarin therapy. These examples highlight some benefits of pharmacogenomics research that is inclusive of AIAN participants and underscores the potential harms of not including a fuller spectrum of genetic variation in pharmacogenetic-guided drug therapy.

The identification of novel variants requires time-intensive validation of genotype-phenotype function in vitro^27^ or in silico^28^ models and validation in independent tribal populations. Additionally, drawing broad conclusions for all Indigenous peoples from basic and clinical research studies involving only a few AIAN populations is problematic as communities may differ. Despite diverse views related to pharmacogenomic clinical utility and cost effectiveness, there is inherent value in doing PGx research with AIAN peoples. Characterizing pharmacogenetic variants in Indigenous populations is an important step to establishing actionable precision drug dosing to improve clinical outcomes. The effect sizes of genome-wide association study loci also show great heterogeneity across different ancestries, and consequently, derived risk prediction scores may not translate well for diverse populations^29^, which necessitates developing comprehensive variant information and improved analysis frameworks with Indigenous communities.

## Community-engaged and collaborative pharmacogenomic research

Community-engaged research approaches, such as community-based participatory research (CBPR), encourage the building of partnerships between communities and researchers, in which research addresses community health priorities and helps build relationships and trust^30^. While there may not be clear or immediate benefits to research, it is important for researchers to try to establish direct or indirect benefits for AIAN peoples at the outset. These benefits could pertain to the individual research participant, but also could emphasize the potential to improve health outcomes for the future generations of AIAN peoples. CBPR approaches in pharmacogenomics research must prioritize community engagement and capacity building. For example, the Northwest-Alaska Pharmacogenomics Research Network established community-academic partnerships with three tribal community partners—and in the example of *CYP2C9* variation and the anticoagulant warfarin described above—they used CBPR approaches that included maintaining tribal oversight, frequent discussions with tribal community advisory boards, and some indirect benefits included the training of Indigenous scholars and community members^31–34^. Community-engaged research approaches are also being used by some private companies, potentially bringing flexibility and more sustainable funding mechanisms^35^. Essential considerations for increasing the inclusion of AIAN communities in precision medicine and pharmacogenomic research are: (1) ensuring tribal governance and oversight—particularly around issues of data sovereignty with respect to biospecimens and data^36^; (2) pursuing research that is inclusive of AIAN values and priorities^4^; (3) establishing productive research partnerships with sustained funding, which is challenging given current funding structures where grants are awarded for only finite periods of time; and (4) shifting toward long-term, community-engaged pharmacogenomics research.

Various community-engaged approaches have been used to develop Indigenous reference genomes, genomic databases, and biobanks^37^; these resources are essential to ensuring Indigenous diversity is represented by improving imputation methods and providing equitable access to genomic research. For example, better reference genomes are needed to reflect the diversity of the world’s populations as well as to improve imputation and read mapping (which influences the apparent frequencies of rare variants or eliminates potential biases)^38^. Importantly, Indigenous communities throughout the world are heterogeneous for novel variants, such that establishing “representative” or reference genomes may not be possible. Two initiatives—“Silent Genomes” in Canada (www.bcchr.ca/silent-genomes-project) and “Aotearoa Variome” in New Zealand (www.genomics-aotearoa.org.nz)—aim to create Indigenous background variant libraries (IBVL) using a CBPR approach. Specifically, the Silent Genomes project is working to create an IBVL with the Indigenous people of Canada. The Aotearoa Variome is sequencing the genomes of New Zealanders, emphasizing Māori and Polynesian peoples. Biospecimen and data storage is also a primary concern for Indigenous people. The Native BioData Consortium, housed on tribal lands in South Dakota, recently formed to store Indigenous biospecimens and associated data (https://nativebio.org). These genomics initiatives are led and designed by Indigenous researchers, further emphasizing the importance of Indigenous knowledge and tribal capacity building skill sets to ensure Indigenous governance over the data^37^.

Pharmacogenomic initiatives with Indigenous populations are global clinical research priorities^1,39–41^, highlighting the need for comprehensive characterization of pharmacogenetic variation to guide precise medication management for Indigenous patients. The translation of pharmacogenomics research into clinical practice has generated much enthusiasm for the possibility to improve outcomes and personalize treatments, yet remains largely unfulfilled for Indigenous communities. Thus, it is imperative to prioritize engagement and collaborations between researchers and healthcare facilities serving Indigenous peoples to ensure inclusion and representation in pharmacogenetic based-precision medicine. Most pharmacogenomic implementation efforts have focused on patients served by large healthcare systems, and more research and resources need to be allocated to promoting clinical decision support, pharmacogenetic testing, and the return of clinically significant results in Indigenous communities.

We are hopeful that the long-term health of AIAN communities can be improved and sustained with community-engaged approaches for pharmacogenomic-based precision medicine inclusive of traditional AIAN values and ethics. By using inclusive and community-driven approaches in pharmacogenomic research, we can diversify knowledge of pharmacogenomic variation and advance clinical implementation aimed at improving health and well-being in AIAN peoples.
